# Enhancing Blue Polymer Light-Emitting Diode Performance by Optimizing the Layer Thickness and the Insertion of a Hole-Transporting Layer

**DOI:** 10.3390/polym16162347

**Published:** 2024-08-20

**Authors:** A. Saad, N. Hamad, Rasul Al Foysal Redoy, Suling Zhao, S. Wageh

**Affiliations:** 1 Department of Physics, College of Science and Humanities in Al-Kharj, Prince Sattam bin Abdulaziz University, Al-Kharj 11942, Saudi Arabian.hamad@psau.edu.sa (N.H.); 2 Department of Physics, Faculty of Science, King Abdulaziz University, Jeddah 21589, Saudi Arabia; rredoy@stu.kau.edu.sa; 3 Key Laboratory of Luminescence and Optical Information, Institute of Optoelectronics Technology, Beijing Jiaotong University, Beijing 100044, China; 4 Physics and Engineering Mathematics Department, Faculty of Electronic Engineering, Menoufia University, Menouf 32952, Egypt

**Keywords:** blue light-emitting diode, BP105, TAPC

## Abstract

Polymer light-emitting diodes (PLEDs) hold immense promise for energy-efficient lighting and full-color display technologies. In particular, blue PLEDs play a pivotal role in achieving color balance and reducing energy consumption. The optimization of layer thickness in these devices is critical for enhancing their efficiency. PLED layer thickness control impacts exciton recombination probability, charge transport efficiency, and optical resonance, influencing light emission properties. However, experimental variations in layer thickness are complex and costly. This study employed simulations to explore the impact of layer thickness variations on the optical and electrical properties of blue light-emitting diodes. Comparing the simulation results with experimental data achieves valuable insights for optimizing the device’s performance. Our findings revealed that controlling the insertion of a layer that works as a hole-transporting and electron-blocking layer (EBL) could greatly enhance the performance of PLEDs. In addition, changing the active layer thickness could optimize device performance. The obtained results in this work contribute to the development of advanced PLED technology and organic light-emitting diodes (OLEDs).

## 1. Introduction

PLEDs, or conjugated polymer light-emitting diodes, are quite popular because of their simple solution procedure and possible high emission efficiency. As such, it is thought that PLEDs will serve as the foundation for the upcoming generation of flat-panel displays [1]. These specialized light-emitting diodes are essential parts of bright, energy-saving lighting and display systems. Blue PLEDs are a crucial invention in the future generation of lighting and visual solutions because they are essential for achieving full-color displays, maintaining color balance, and helping to minimize energy usage. Scientists and engineers have been working hard over the years to increase the efficiency of blue PLEDs, which will further qualify them for various uses. Controlling the layer thickness in PLEDs is an essential area of research for improving LED performance. The thickness of the organic layers in a PLED influences the probability of excitons (electron–hole pairs) recombining and the emission of light [2]. An optimal layer thickness ensures that a higher percentage of excitons recombine to produce light rather than being lost through nonradiative processes.

Consequently, controlling layer thickness could enhance light emission efficiency [3]. A well-tuned thickness can reduce resistive losses and increase the device’s overall efficiency by facilitating more efficient charge transport. It is possible to generate optical resonance by varying the thickness of some layers, such as the emissive layer [4,5]. More effective light emission and a decrease in optical losses can be achieved by optimizing light confinement and extraction through this resonance [6]. Light emission in full-color displays can be controlled by varying the emissive layer thickness. Achieving a wide color spectrum and accurate color representation requires this fine control. However, studying the impact of different thicknesses presents challenges, as achieving such variations necessitates complex and costly deposition methods. Therefore, conducting simulations emerges as the preferable choice prior to experimental endeavors. Simulations offer a more cost-effective and efficient means of exploring the effects of layer thickness variations.

Generally, light-emitting devices consist of organic structures mainly based on the dual injection of holes and electrons from two electrodes with opposite charges. The injected charges should finally enter the emissive layer to form an exciton followed by light emission. Consequently, to enhance the device performance, multilayer structures should be preferred, including a separate hole-transport layer (HTL), electron-transport layer, and emissive layer (EML). The role of the HTL layer is to enhance the hole injection into the active layer, block the electron from overflowing, and confine the formed exciton in the active layer. Accordingly, the insertion of the HTL is a preferable choice for increasing the OLED efficiency.

In polymer light-emitting diode (PLED) research, running simulations in addition to conventional experimental work provides an economical, effective, and adaptable way to investigate a broad range of layer thickness scenarios. Through the exploration of unfeasible or extreme scenarios and the quick refinement of hypotheses, simulations maximize resource use and reduce risk. They help identify suitable experimental candidates by offering predictive insight into the behavior of layer thickness. Furthermore, simulations facilitate parametric analysis and expedite the experimentation procedure. Simulations supplement experimental research by addressing safety and environmental concerns, eliminating material waste, and testing hypotheses. This helps to advance the development of cutting-edge PLED technology and improves our understanding of PLED behavior.

Besides the importance of blue light-emitting devices (OLEDs) for application in flat panel displays and solid-state lighting [7], blue light has important therapeutic applications, including treating acne breakouts, psoriasis, seasonal affective disorders, and newborn jaundice [8]. Also, blue LEDs have applications in food safety, such as tomato treatment; blue light can delay ripening and, thereby, extend the storage life of tomatoes [9,10].

According to the abovementioned requirements, this paper introduces a simulation and the optimization of a blue light-emitting diode based on BP105 as an emission layer. The analysis of optical and electrical properties concerning changes in the active and electron-blocking layers’ thickness was investigated. To conduct our simulations, we employed the commercially available software, Setfos v5.4, developed by Fluxim [11]. Utilizing this software, we successfully simulated experimental data for the blue multilayer polymer light-emitting diode, consisting of ITO, PEDOT: PSS, BP105, and LiF/Ca/Al [12,13]. After confirming the validity of the simulation method, we optimized the performance of the device by varying the thickness of the active layer. In addition, another design with the insertion of a layer that works simultaneously as HTL and electron blocking was investigated and optimized.

The selection of the PLED architecture as ITO, PEDOT: PSS, BP105, and LiF/Ca/Al arising from this arrangement has a good opportunity for further advancement and innovation in the optoelectronics industry. This arrangement combines the advantages of solution-processable materials such as PEDOT:PSS and BP105, creating a promising platform for cost-effective and scalable manufacturing. In this arrangement, the combination of LiF/Ca/Al was selected as one of the two electrodes. The LiF/Ca/Al electrode has excellent electrical performance and exhibits a low turn-on voltage. Furthermore, ITO, PEDOT: PSS, BP105, and LiF/Ca/Al designs have low heat generation and power consumption, thereby extending their lifespan. Moreover, the device produces high brightness through effective charge injection and transport systems.

## 2. Numerical Simulation of the Experimental Model

**Figure 1:**
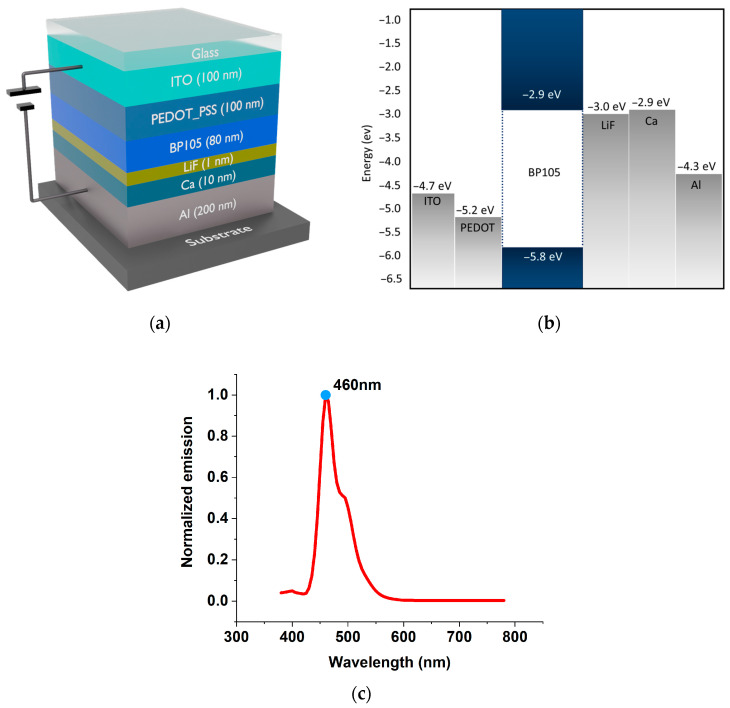
(**a**) Layer structure. (**b**) Band energy diagram. (**c**) Emission of BP105.

A drift-diffusion technique, which has been published in numerous other publications [14,15,16], serves as the foundation for the electrical simulations used in this study. The measured curves from the electrical experiments can be replicated by solving the equations in the steady-state, transient, and frequency domains. The computed exciton densities are fed into the optical solver for electro-optical simulations based on the transfer matrix and dipole emission models [17,18]. The required inputs for the simulation are the layer sequence with respective thicknesses, refractive indices, and each layer’s electrical and excitonic material parameters. The results obtained in references [12,13] were simulated to confirm our computation’s validity. The structure and the layer thicknesses of the blue light emitting device are as follows: ITO(100 nm), PEDOT: PSS(100 nm), BP105(80 nm), LiF(1 nm), Ca(10 nm), and Al(200 nm) [12,13]. The schematic construction of the device and band energy diagram are shown in Figure 1a,b. In this device, the active layer consists of a blue-emitting polymer known as BP105, which was developed by The Dow Chemical Company (Midland, MI, USA). The emission of BP105 is displayed in Figure 1c.

BP105, along with the top and bottom electrodes, acts as the main emission layer. ITO/PEDOT:PSS works as the top electrode (anode). ITO is a well-known transparent electrode with a work function of −4.7 eV. However, the potential barrier is large between the work function of ITO and the HOMO level of BP105, causing inefficient carrier injection. A hybrid electrode of ITO/PEDOT:PSS was introduced to lower this barrier. Comparatively to ITO alone (−4.7 eV), PEDOT’s intrinsic work function (−5.2 eV) is closer to the HOMO level of BP105 (−5.8 eV). PEDOT:PSS molecules have intrinsic dipole moments due to their chemical structure. Variations in their work functions and Fermi levels may cause electrons to migrate between ITO and PEDOT:PSS when PEDOT:PSS is deposited on ITO. This electron movement builds both positive and negative charges at the contact, creating an electric dipole layer. The vacuum level is normally shifted upward by the development of this dipole layer at the ITO/PEDOT:PSS interface, raising the combined system’s work function. This lowers the energy barrier for hole injection into the active layer by bringing the anode’s effective work function closer to the HOMO level of BP105. PEDOT:PSS also provides a smoother and more consistent surface than the rough bare surface of ITO. Reducing the number of interface defects helps improve surface quality, thereby facilitating better electrical and physical contact with BP105.

For better performance, LED requires an electrode for the cathode that has a lower work function and is in good alignment with the LUMO level of BP105. For that, a combined electrode of LiF/Ca/Al was introduced to the structure. LiF has a very wide bandgap (~14 eV), which makes it an excellent insulator. Despite being an insulator, a very thin layer of LiF (1 nm) was used as a buffer layer to reduce any interface recombination and improve carrier confinement. However, the work function of LiF (−3.0 eV) and the LUMO level of BP105 still create a potential barrier. Ca was included to lower this barrier. The LUMO level of BP105 is well-aligned with the comparatively low work function of calcium, which is approximately −2.9 eV. The work function of the integrated system decreases when calcium deposits on LiF. This work function modification is crucial for aligning the energy levels between the cathode and the active layer, reducing the barrier for electron injection. However, calcium is a highly reactive metal that can trigger device degradation very quickly. A layer of aluminum on top of calcium can improve both the device’s stability and carrier injections. Aluminum was chosen for its excellent electrical conductivity and its stability. Aluminum has a work function of about −4.3 eV. Although this is higher than that of calcium, the combination of LiF and Ca layers ensures that the overall injection barrier is minimized.

The optical and electrical parameters, including refractive index, HOMO-LUMO energy levels, charge mobility, and radiative and nonradiative decay rates of the materials, were applied from the built-in material database integrated into Setfos. The carrier mobility of BP105 was determined using the Extended Gaussian Disorder Model (EGDM). This model is widely employed to describe charge transport in organic materials, providing valuable insights into the mobility of charge carriers within the BP105 active layer of the device [19]. An Exponential Density of States (DOS) model was utilized to analyze the trap state. Within this model, the trapping density of electrons was set to 2 × 10^24^ m^−3^ [19]. This model allowed for a comprehensive investigation of electron trapping phenomena, providing a better understanding of the device’s behavior. Recombination processes in the device were modeled using the Langevin model, where the recombination efficiency was specifically set to 1. The Langevin model is commonly used to describe the recombination of charge carriers within semiconductor materials and organic devices. In this case, the efficiency value of 1 suggests that all potential recombination events were considered without any reduction or losses, resulting in a comprehensive analysis of recombination processes. The simulations were conducted at a temperature of 293 Kelvin (293 K). Furthermore, in the simulations, the device was surrounded by glass, a substrate, and air, as illustrated in Figure 1a. These surrounding materials and conditions were integrated into the simulation setup to closely replicate the real-world environment in which the optoelectronic device operates.

**Figure 2:**
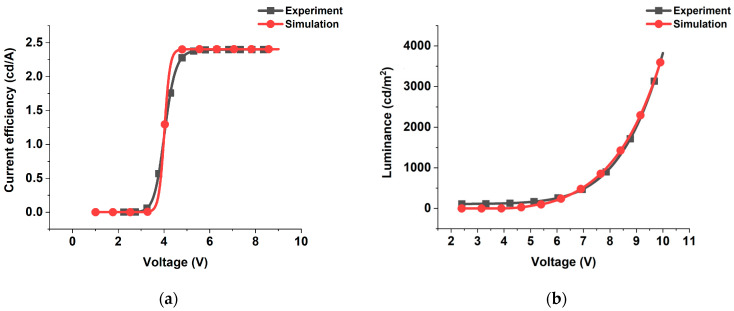
Validation of simulation results against experimental data for blue polymer light-emitting diode performance. (**a**) Current efficiency; (**b**) luminance.

The simulation of current efficiency and luminance of the device consisting of ITO(100 nm), PEDOT: PSS(100 nm), BP105(80 nm), LiF(1 nm), Ca(10 nm), Al(200 nm) are shown in Figure 2. In addition, the experimental data of similar construction published in references [12,13] are displayed to confirm the validity of our method. Clearly, the simulation closely matched the experimental results, affirming its accuracy and suitability in scientific contexts.

## 3. Device Optimization

### 3.1. Varying Thickness of Active Layer

In the previous section, we demonstrated the ability of our simulations to accurately reproduce the experimental characterizations of the BP105 blue light-emitting polymer. Our modeling approach enables the simultaneous variation of parameters within the PLED stack and the scattering layer. Therefore, in this section, we present how the device can be further optimized.

**Figure 3:**
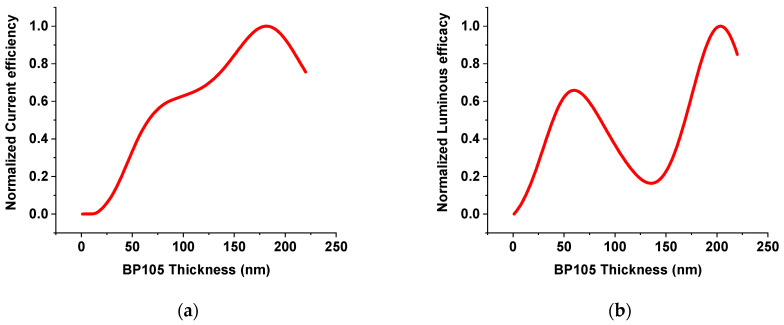
The effect of varying the active layer of BP105 thickness on (**a**) current efficiency; and (**b**) luminous efficacy.

The performance of the light-emitting diode was first optimized by varying the thickness of the emission layer. The effect of varying active layer thickness was investigated while keeping the other layer thicknesses constant. Figure 3a,b show the effect of changing BP105 thickness on the current efficiency and luminous efficacy with an applied voltage of 5 V. Increasing the thickness from 10 nm to 80 nm continuously causes an increase in current efficiency, and then the current efficiency has the first maximum value in the range of the thickness from 80 to 120 nm. Further, increasing thickness beyond 120 nm causes an increase in current efficiency, reaching the highest maximum at 180 nm thickness. The behavior for luminous efficacy with varying the thickness of the active layer showed two maximums, one at the thickness of the active layer of 60 nm and the second at the thickness of the active layer of 200 nm.

**Figure 4:**
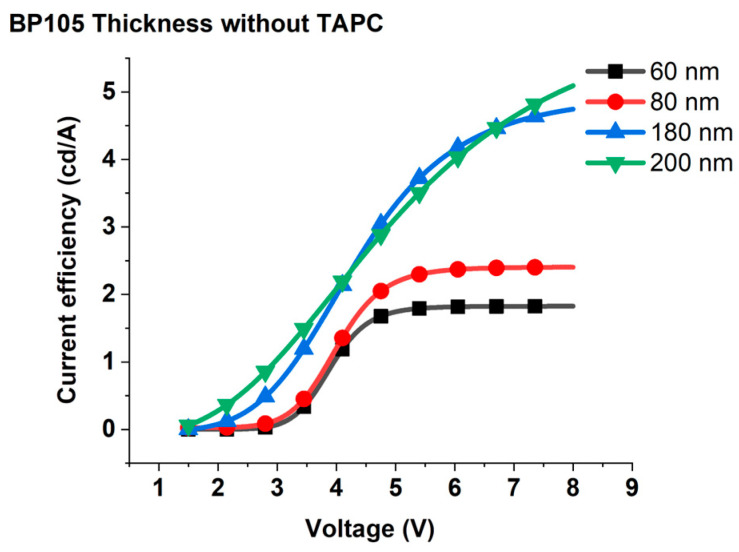
Current efficiency for different thicknesses of the emission layer of the device consists of ITO(100 nm), PEDOT: PSS(100 nm), BP105(60, 80, 180, and 200 nm), LiF(1 nm), Ca(10 nm), and Al(200 nm).

According to the investigation of the device with various thicknesses of BP105, we found that the thicknesses of 60 nm, 80 nm, 180 nm, and 200 nm are important thicknesses of the active layer to study. Consequently, we investigated the current efficiency and luminous efficacy against voltage variation for the devices of BP105 with thicknesses of 60 nm, 80 nm, 180 nm, and 200 nm. Figure 4 shows the effect of variation of applied voltage on the current efficiency for different thicknesses of the emission layer.

**Figure 5:**
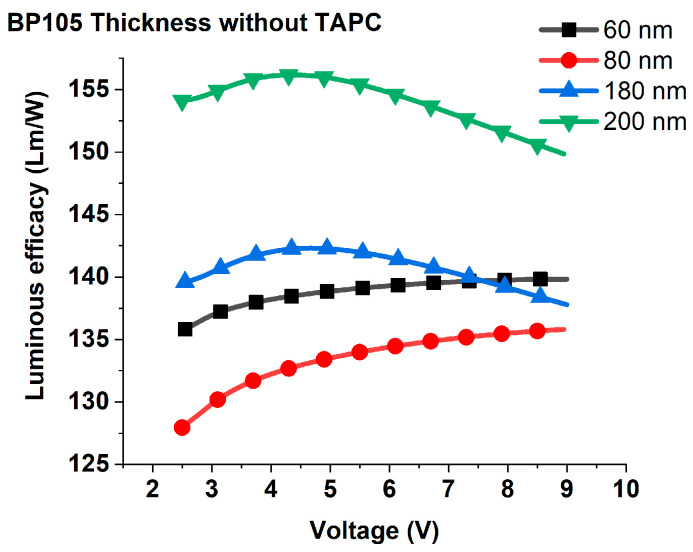
Luminous efficacy for different thicknesses of the emission layer.

The increase in applied voltage leads to an increase in current efficiency continuously up to 5 V; beyond 5 V, the efficiency tends to saturate. As the voltage is increased with low values, the electric field becomes stronger, which facilitates the injection of more charge carriers into the device and, in consequence, causes an increase in current efficiency. While the saturation of current at higher voltage values can be ascribed to one of the following reasons: (1) there can be a mismatch between the densities of electrons and holes injected into the active layer. This charge imbalance can result in inefficient recombination processes, where excess carriers remain unpaired and contribute to nonradiative recombination channels. Consequently, the current efficiency may be saturate at higher voltage [20]. (2) At higher voltages, the density of charge carriers (electrons and holes) within the OLED increases. This elevated carrier density can lead to increased exciton-quenching effects, where excitons (electron-hole pairs) are more likely to undergo nonradiative decay processes, such as collisional quenching or exciton–exciton annihilation, rather than radiative recombination to emit light leads to current saturation [21]. To decide which mechanism is responsible for current saturation at higher voltage, we investigated the effect of applied voltage on the luminous efficacy. Figure 5 shows luminous efficacy against voltage variation for various devices that possess different thicknesses of active layer. Clearly, the luminous efficacy started to decrease at higher voltage, which indicates that the second mechanism is responsible for the saturation of current efficiency. At higher voltages, the process of collisional quenching or exciton–exciton annihilation and nonradiative recombination causes a decrease in the efficiency of converting injected charges into emitted photons and consequently, luminous efficacy decreases.

Increasing the thickness of active layers from 60 nm to 180 nm and 200 nm causes an enhancement of the current efficiency and luminous efficacy; these results can be attributed to the increasing thickness of Bp105 facilitating a higher density of polymer chains and a greater volume for exciton formation. This can increase the probability of exciton generation per unit area, leading to more efficient light emission and improved luminous efficacy. Furthermore, large thicknesses can help optimize the distribution and density of charge carriers within the device, leading to improved charge balance [22]. This can minimize nonradiative recombination processes and enhance light emission efficiency, thereby increasing luminous efficacy. Conversely, with the increasing thickness from 60 nm to 80 nm, the current efficiency increases, but luminous efficacy decreases. These results can be explained as follows. The thickness of 80 nm may exacerbate optical losses within the device due to weak microcavity effects (i.e., non-resonance optical length) [23]. This loss can reduce the amount of light extracted from the device and diminish luminous efficacy, even if the current efficiency improves.

### 3.2. Effect of Insertion of HTL Layer on the Performance of the OLED

**Figure 6:**
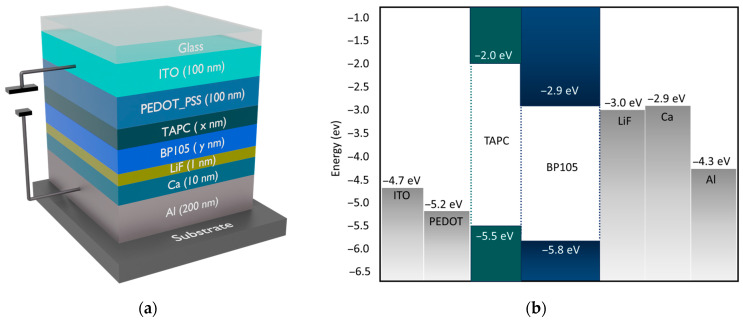
Layer structure and band energy diagram after adding TAPC. (**a**) Layer structure; (**b**) band energy diagram.

Another strategy applied to improve the performance of the device is the introduction of TAPC (1,1-bis[(di-4-tolylamino)phenyl]cyclohexane) as an intermediary layer between PEDOT: PSS and the active layer. Due to the high triplet energy (2.9 eV) and LUMO levels (−2.0 eV) of TAPC, it works as a hole-transporting and electron-blocking layer. The schematic construction of the device with the insertion of the TAPC layer along with the band energy diagram is shown in Figure 6.

**Figure 7:**
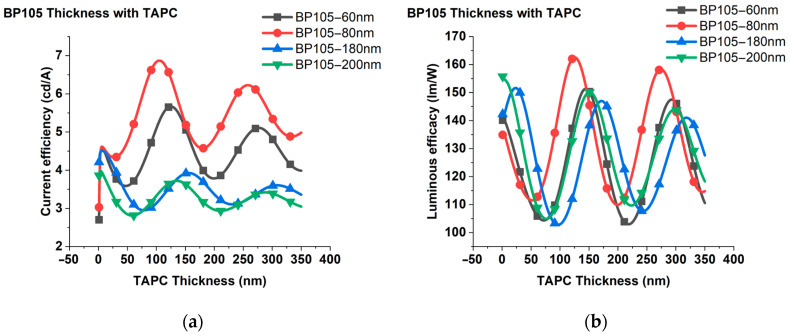
Varying the thicknesses of TAPC from 1 nm to 350 nm for the thicknesses of 60, 80, 180, and 200 nm of the BP105 layer. (**a**) Current efficiency; (**b**) luminous efficacy.

The changes in current efficiency and luminous efficacy with varying thicknesses of TAPC from 1 nm to 350 nm for the thicknesses of 60, 80, 180, and 200 nm of the BP105 layer are shown in Figure 7a,b. Obviously, three maximums appeared that achieve optimum current efficiency, which varied for different thicknesses of the active layer. Similar behavior was obtained for luminous efficacy. According to the appeared maximums with varying TAPC thicknesses, we investigated the performance of various devices with thicknesses of 60 nm, 80 nm, 180 nm, and 200 nm for the BP105 layer.

**Figure 8:**
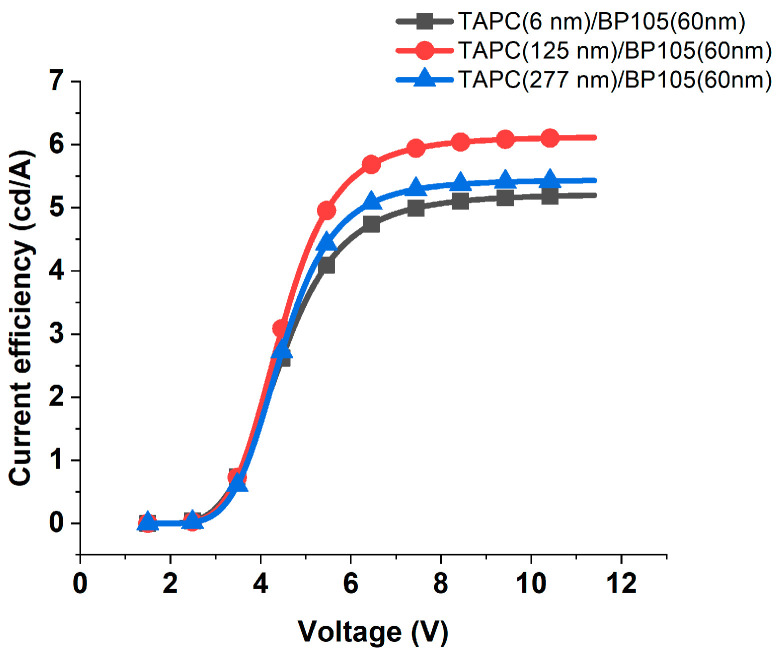
Current efficiency against the applied voltage for the device with 60 nm of BP105, with various thicknesses of TAPC hole-transporting layer thicknesses (6 nm, 125 nm, and 277 nm).

The effects of varying the thicknesses of TAPC on current efficiency and luminous efficacy at different applied voltages for the four devices with BP105 of thicknesses of 60 nm, 80 nm, 180 nm, and 200 nm were investigated. Figure 8 shows the current efficiency against the applied voltage for the device consisting of ITO(100 nm), PEDOT: PSS(100 nm), TAPC (x), BP105(60 nm), LiF(1 nm), Ca(10 nm), and Al(200 nm), with various thicknesses of TAPC hole-transporting layer thicknesses (6 nm, 125 nm, and 277 nm). At low voltage, the current efficiency was very small due to inefficient charge injection. As the voltage increased beyond the threshold (~3.5 V) up to nearly 6 V, the current efficiency rose as more charge carriers were injected and contributed to radiative recombination, and consequently, the current efficiency continuously increased. At higher voltages, the current efficiency was saturated, i.e., limiting the efficiency of converting injected charge carriers into emitted photons, leading to diminished current efficiency despite increasing voltage. The effect of variation of thickness at low voltages below 5 V on the current efficiency was insignificant, but for higher voltages and in the saturation region, applying the thickness of 125 nm, which is compatible with the second maximum, caused an increase in the current efficiency. Conversely, applying the thickness compatible with the third maximum of 277 nm, the current efficiency increased relative to the thickness of 6 nm and decreased relative to the thickness of 125 nm.

**Figure 9:**
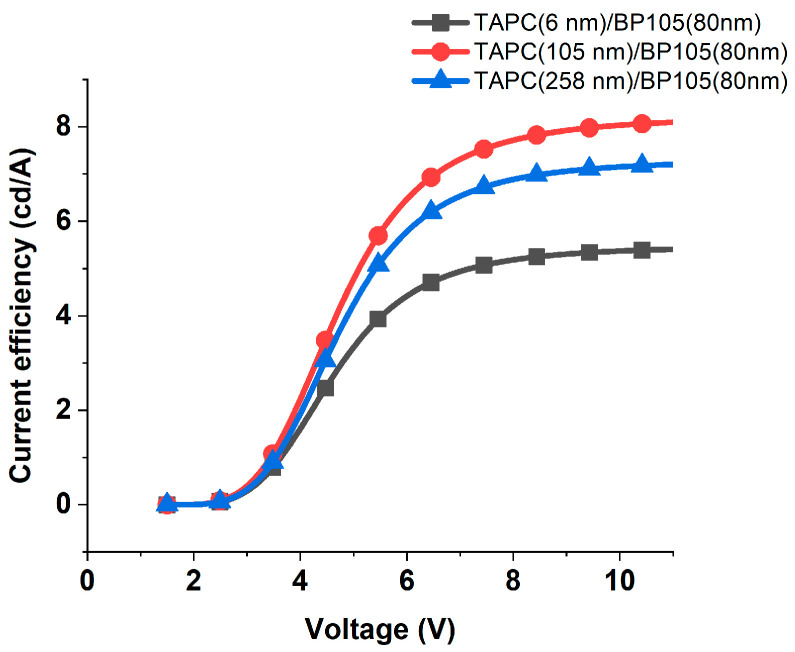
Current efficiency against the applied voltage for the device with 80 nm of BP105, with various thicknesses of TAPC hole-transporting layer thicknesses (6 nm, 105 nm, and 258 nm).

The behavior for the device with ITO(100 nm), PEDOT: PSS(100 nm), TAPC (x), BP105(80 nm), LiF(1 nm), Ca(10 nm), and Al(200 nm) and various TAPC hole-transporting layer thicknesses was similar to the device with BP105 of 60 nm but with an increase in current efficiency, as shown in Figure 9. We should keep in mind that the thickness that was given the highest values of luminous efficacy does not equal the thickness that has given higher current efficiency.

**Figure 10:**
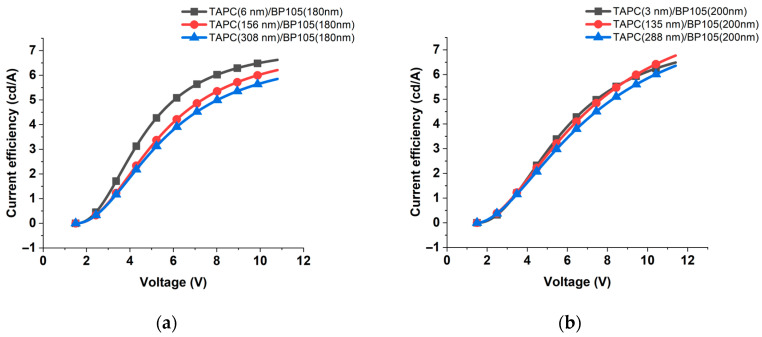
Current efficiency against the applied voltage for the device with (**a**) 180 nm and (**b**) 200 nm of BP105, with various thicknesses of TAPC hole-transporting layer thicknesses.

The behavior for devices with higher thicknesses of 180 nm and 200 nm of the active layer with the variation of TAPC thicknesses are different, as shown in Figure 10a,b. The current efficiency continuously increased and did not reach complete saturation. In addition, the current efficiency maximum values were less than those obtained for the devices with thinner active layers. These could be explained by the fact that the thinner layers lead to more efficient charge transport and reduced exciton quenching at the interfaces, resulting in higher current efficiency.

**Figure 11:**
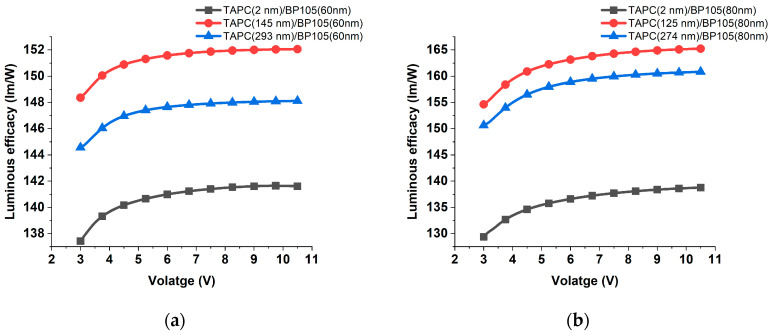

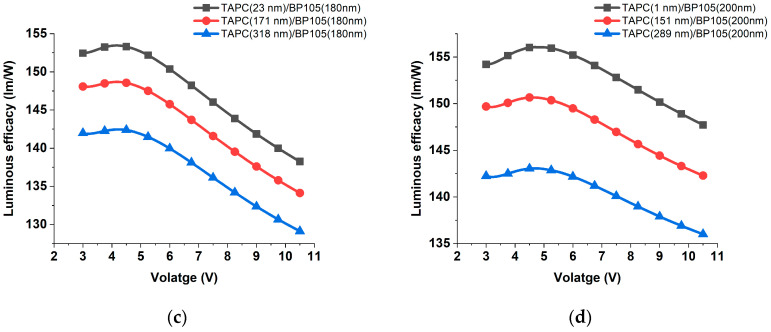
Luminous efficacy against the applied voltage for the device with (**a**) 60 nm, (**b**) 80 nm, (**c**) 180 nm, and (**d**) 200 nm of BP105, with various thicknesses of TAPC hole-transporting layer thicknesses.

The effect of variation of TAPC thicknesses on the luminous efficacy of the devices with different thicknesses of active layer BP105 was investigated. The luminous efficacy for the devices with thicknesses of the active layer of 60 and 80 nm and various thicknesses of TAPC are shown in Figure 11a,b. The variation of TAPC layer thickness for the two devices showed similar behaviors but with the enhancement of efficacy for 80 nm of BP105. For the devices with a large active layer thickness of 180 nm and 200 nm, the trend of evolution of luminous efficacy in comparison with thinner devices was different. At low voltage, the luminous efficacy increased to a maximum, followed by a pronounced decrease at higher voltages, as shown in Figure 11c,d. These results could be attributed to the fact that there is an exciton quenching or non-radiative recombination process for the thicker active layer due to an imbalance in charge transportation within the device, leading to non-uniform current distribution and localized regions of high current density [24].

**Figure 12:**
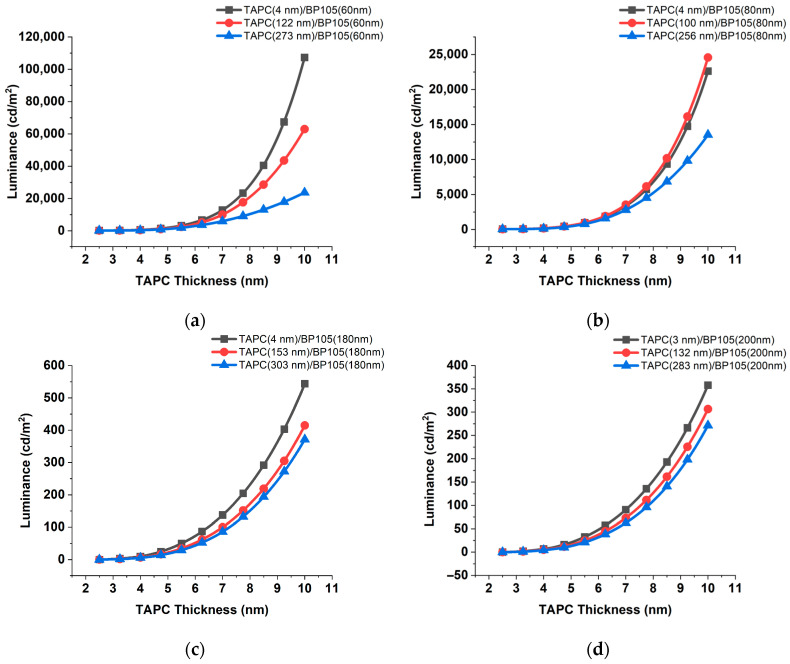
Luminance versus voltage for devices with different TAPC thicknesses for devices with a thickness of BP105 of (**a**) 60 nm, (**b**) 80 nm, (**c**) 180 nm, and (**d**) 200 nm.

The luminance of four devices with a thickness of the active layer of 60 nm, 80 nm, 180 nm, and 200 nm with varying thicknesses of the TAPC hole-transporting layer was investigated, as shown in Figure 12a–d. The luminance slowly increased at a lower voltage, reaching the built-in voltage value followed by an abrupt increase. Herein, we have a two-dimensional investigation. The first is the effect of changing hole-transporting thickness, and the second is the variation of the thickness of the active layer. For the diodes with thicknesses of active layers 60, 180, and 200 nm, the increase in hole-transporting layer thickness showed a pronounced decrease in luminance. However, the behavior of the device with an active layer thickness of 80 nm was different, and the maximum luminance obtained for the device with the thickness of TAPC was 100 nm. It is worth mentioning that the devices with the sum of the thicknesses of the hole-transporting layer and the active layer were close to 60, or multiple of them (i.e., the sum of HTL + EML = 180 nm) yielded the optimum luminance values. In addition, the luminance of devices with the sum of the small thicknesses of HTL + EML achieved higher luminance in comparison with the devices of large values of the sum of the thicknesses of HTL + EML. The decrease in luminance with thicker HTL can be produced due to the following factors: (1) hindered injection efficiency of holes into the emissive layer, leading to an imbalance in charge carriers; (2) exciton quenching at the interface between the HTL and the emissive layer; (3) the weak microcavity effect; and (4) thicker HTL layers resulting in more absorption of emitted light within the device, reducing the amount of light that escapes the device. The fourth reason can be excluded due to the absorption of TAPC located at higher energy relative to the emission wavelength of the device. As the devices with multiple thicknesses of HTL + EML achieve the optimum luminance, we can conclude that the main reasons that lead to a decrease in the luminance are the combination of the non-resonance thickness of microcavity and imbalance in charge carriers.

### 3.3. Comparison of the Performance of the PLED with and without TAPC

**Figure 13:**
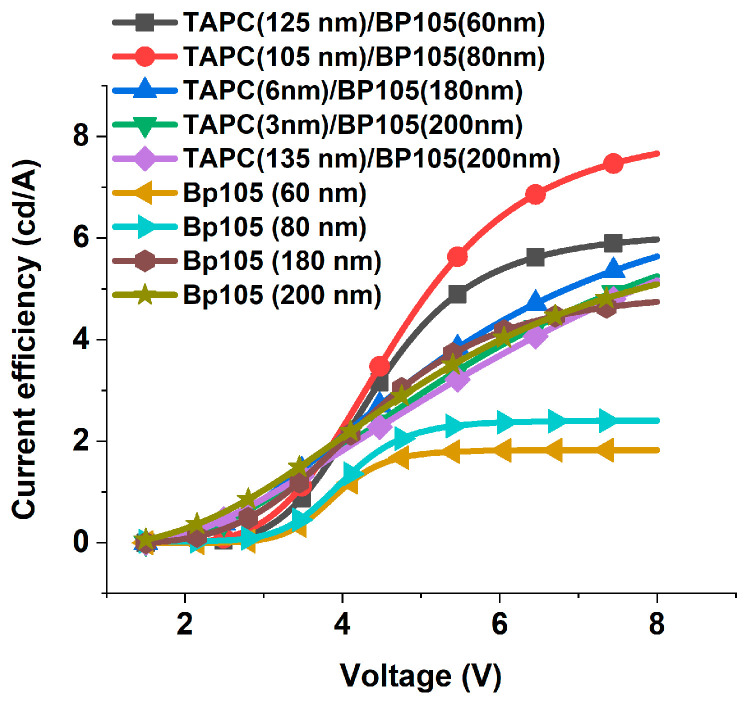
Comparison of the current efficiency of devices with different combinations of TAPC and BP105.

In the previous sections, we detected the effective thickness of the active layer and the hole-transporting layer and their influence on the performance of the solar cell (Table 1). In this section, we will compare their performance. Figure 13 shows the current efficiency of devices with different combinations of TAPC and BP105. Comparing the current efficiency for the devices with and without the TAPC hole-transporting layer showed that the devices with the sum of the thickness HTL + EML or single EML close to 180 nm achieved the optimum current efficiency. The devices with the TAPC layer achieved the highest current efficiency relative to the devices without HTL. These results show that the improvement of hole injection, blocking electrons, and balance of carriers are the main factors that affect the current efficiency.

**Figure 14:**
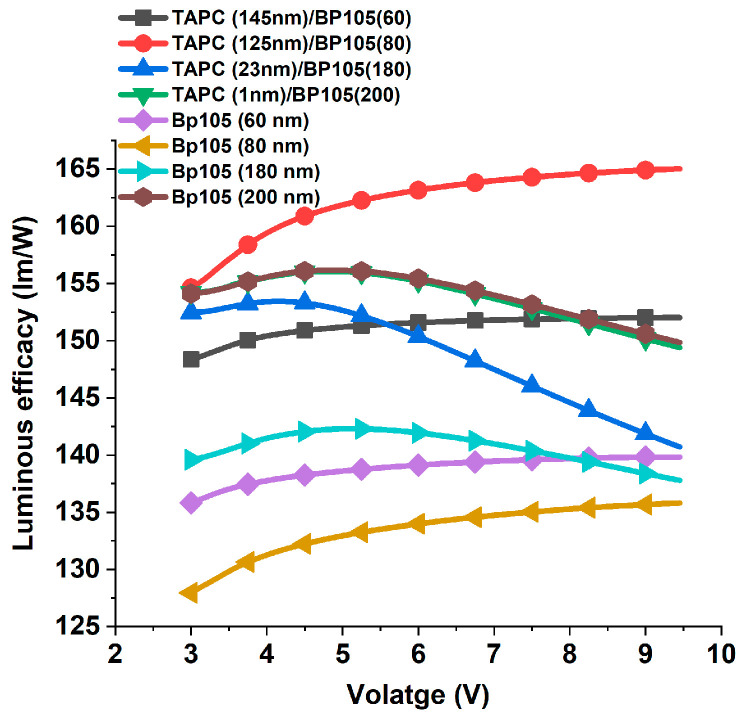
Comparison of the luminous efficacy of devices with different combinations of TAPC and BP105.

Figure 14 shows the comparison of the luminous efficacy of the devices with and without TAPC. Unlike current efficiency, the device with the sum of the thicknesses of BP105 and TAPC close to any specific thickness does not achieve the optimum luminous efficacy. Instead, the active layer thickness of 80 nm achieved the highest luminous efficacy, exceeding the similar device without TAPC at 4.5 V by about 21.66%. Although the active layer thickness less than or equal to 80 nm showed an increasing trend, the devices that have a thickness greater than or equal to 180 nm showed different behavior at a higher voltage. The luminous efficacy decreased at higher voltages for thicker devices.

**Figure 15:**
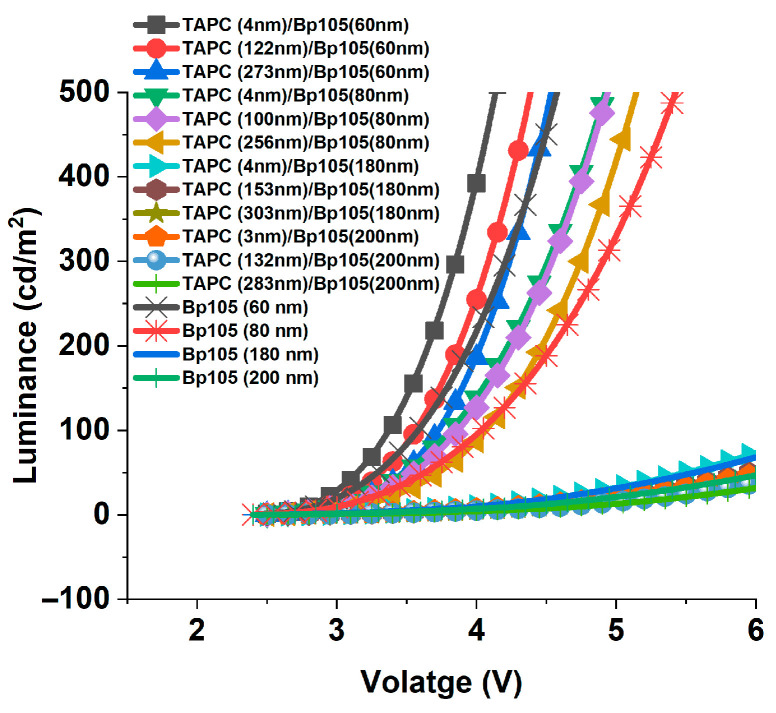
Comparison of the luminance of devices with different combinations of TAPC and BP105.

A comparison of the luminance of the devices that included HTL showed a pronounced improvement relative to the devices without HTL (Figure 15). The luminance increased nearly two-fold for the PLED included TAPC relative to that one without HTL.

Astonishing results for the devices with higher thicknesses of TAPC of 273 nm and BP105 (60 nm) show that the change in luminance is negligible. These results mean that the factors that lead to the enhancement of luminance are comparable with factors that lead to decreasing the luminance at this thickness of TAPC.

## 4. Conclusions

In this study, we conducted a comprehensive simulation of a blue light-emitting polymer device, focusing on the impact of an electron-blocking layer (EBL), the thickness variation in both the EBL and active layer (Bp105), on the device’s optical and electrical performance. Our findings provide valuable insights into the enhancement of the device’s efficiency and demonstrate the critical role of EBL thickness in optimizing the performance of blue light-emitting polymers.

Our investigation revealed that the incorporation of hole transporting and an electron-blocking layer significantly improved the overall performance of the device. This improvement can be attributed to the EBL’s ability to confine electrons within the active layer, preventing them from escaping the device and reducing recombination losses.

Furthermore, we explored the impact of varying the active layer thickness (Bp105) combined with hole-transporting layer thickness and found that an active layer thickness of approximately 80 nanometers (nm) and a TAPC of 125 nm led to the best performance of luminance efficacy. This thickness optimization likely arises from the balance between efficient charge carrier transport and recombination probability within the active layer.

In summary, our study underscores the importance of the electron-blocking layer in enhancing the optical and electrical performance of blue light-emitting polymer devices. By fine-tuning the thickness of the EBL and active layer, we can achieve the best possible device performance. These findings have significant implications for the design and development of next-generation optoelectronic devices and hold promise for the advancement of organic light-emitting diodes (OLEDs) and other related technologies. Future research could further investigate the underlying mechanisms behind these enhancements and explore additional strategies to improve device efficiency and stability.

## Figures and Tables

**Table 1:** Overview of every peak after thickness variation of BP105 from 1 nm to 225 nm and TAPC from 1 nm to 350 nm.

BP105 Thickness (Peaks)		TAPC Thickness (Peaks)		
		Current Efficiency	Luminous Efficacy	Luminance
Current efficiency	80 nm	6 nm	2 nm	4 nm
		105 nm *	125 nm *	100 nm *
		258 nm	274 nm	256 nm
	180 nm *	6 nm *	23 nm *	4 nm *
		156 nm	171 nm	153 nm
		308 nm	318 nm	303 nm
Luminous efficacy	60 nm	6 nm	2 nm	4 nm *
		125 nm *	145 nm *	122 nm
		277 nm	293 nm	273 nm
	200 nm *	3 nm *	1 nm *	3 nm *
		135 nm	151 nm	132 nm
		288 nm	289 nm	283 nm

Here, * represents the highest peak.

## Data Availability

All data generated or analyzed during this study are included in this article.

## List of Abbreviations

OLED	Organic Light-Emitting Diode
HTL	Hole-transport Layer
ETL	Electron Transport Layer
EML	Emission Layer
EGDM	Extended Gaussian Disorder Model
DOS	Density of States
ITO	Indium Tin Oxide
PEDOT:PSS	Poly(3,4-ethylenedioxythiophene) polystyrene sulfonate
TAPC	1,1-bis[(di-4-tolylamino)phenyl]cyclohexane (a common HTL material)
BP105	A specific blue-emitting polymer
LiF	Lithium Fluoride
Ca	Calcium
Al	Aluminum
